# Associations of Body Mass Index with Sexual Risk-Taking and Injection Drug Use among US High School Students

**DOI:** 10.1155/2014/816071

**Published:** 2014-07-01

**Authors:** Richard Lowry, Leah Robin, Laura Kann, Deborah A. Galuska

**Affiliations:** ^1^Division of Adolescent and School Health, National Center for HIV/AIDS, Viral Hepatitis, STD, and TB Prevention, Centers for Disease Control and Prevention, Atlanta, GA 30329, USA; ^2^Division of Nutrition, Physical Activity, and Obesity, National Center for Chronic Disease Prevention and Health Promotion, Centers for Disease Control and Prevention, Atlanta, GA 30341, USA

## Abstract

The purpose of this study was to determine if body mass index (BMI) is associated with behaviors that may increase risk for HIV and other sexually transmitted diseases (STDs) among US high school students. We analyzed nationally representative data from the 2005–2011 national Youth Risk Behavior Surveys (YRBS) to examine associations of BMI categories with sexual risk behaviors and injection drug use among sexually active high school students, using sex-stratified logistic regression models. Controlling for race/ethnicity and grade, among female and male students, both underweight (BMI < 5th percentile) and obesity (BMI ≥ 95th percentile) were associated with decreased odds of being currently sexually active (i.e., having had sexual intercourse during the past 3 months). However, among sexually active female students, obese females were more likely than normal weight females to have had 4 or more sex partners (odds ratio, OR = 1.59), not used a condom at last sexual intercourse (OR = 1.30), and injected illegal drugs (OR = 1.98). Among sexually active male students, overweight (85th percentile ≤ BMI < 95th percentile) was associated with not using a condom at last sexual intercourse (OR = 1.19) and obesity was associated with injection drug use (OR = 1.42). Among sexually active students, overweight and obesity may be indicators of increased risk for HIV and other STDs.

## 1. Introduction

The public health impact of both childhood obesity and current rates of HIV infection and other sexually transmitted diseases (STDs) among adolescents is substantial, and each problem represents a significant threat to the health of young people in the United States. Approximately one in three US adolescents is either overweight or obese [1]. In addition to increased risk for cardiovascular diseases, type 2 diabetes, and other serious health problems [2, 3], overweight and obesity in childhood are often associated with psychosocial dysfunction, including low self-esteem, depression, and increased suicide ideation and attempts [2, 4–7]. In 2010, young people of ages 13–24 accounted for 26% of all new HIV infections in the USA [8], and nearly half of the 19.7 million new STDs reported each year are among young people of ages 15–24 [9]. Findings in the literature suggest the possibility that obesity, or perhaps body mass index (BMI) more generally, may be related to sexual and nonsexual behaviors that increase risk for HIV and other STDs.

Several possible mechanisms exist whereby childhood obesity may be associated with sexual risk-taking and injection drug use among youth. For example, depression and suicidal thoughts and behaviors experienced by many overweight and obese adolescents may limit their ability or willingness to advocate for sexually responsible behavior in their relationships [10, 11]. Also, one study found that the type of health-risk behavior most strongly associated with suicide attempts among both female and male US high school students was injection drug use [12]. Another possible mechanism for associations between obesity and sexual risk-taking involves early initiation of sexual intercourse. Childhood obesity is associated with early sexual maturation and menarche among girls and increased stature during puberty among girls and boys [2, 13–15]. As a result, overweight and obese youth may appear more physically mature and therefore older than their true age. Among girls and boys, early sexual initiation appears to cluster with other problem behaviors and has been associated with risky sexual behaviors [16–18], substance abuse [16], and suicidal thoughts and behaviors [12, 19]. In addition, children who experience sexual initiation as preteens are often the victims of sexual abuse. Survivors of childhood sexual abuse and nonconsensual sex are at increased risk for a wide range of medical, psychological, and behavioral disorders [20, 21], including sexual risk-taking [22, 23], substance abuse [21, 24], suicidal thoughts and behaviors [21, 25], and obesity, especially among females [26–28]. Finally, it is possible that use of alcohol or drugs before sex may mediate an association between obesity and HIV/STD-related risk behaviors. Although some studies demonstrate no relationship between BMI and sexual experience, one study of extreme obesity among US high school students found that, compared to normal weight female students, female students with extreme obesity had lower odds of ever having sexual intercourse but, once sexually active, had greater odds of drinking alcohol or using drugs before their last sexual encounter [29]. A study of homeless youth in Los Angeles County found that condom use was significantly less likely when hard drug use preceded sex and marginally less likely when heavier drinking preceded sex [30].

Only a small number of studies characterize the relationship between BMI and sexual and nonsexual behaviors that increase risk for HIV and other STDs. Unfortunately, nearly all of these studies focus on overweight and obese youth and exclude underweight youth. Among adolescent and young adult females, some studies have found that being overweight or obese was associated with a reduced likelihood of ever having sexual intercourse [29, 31–34]. However, other studies of adolescent and young adult females have found that being overweight or obese was associated with an increased likelihood of ever having anal sexual intercourse [31], drinking or using drugs before sexual intercourse [29, 35], having 3 or more lifetime partners [36], and having a casual sexual partner [35], as well as a decreased likelihood of using condoms and contraception [36]. The few studies examining the relationship between BMI and sexual risk behaviors among adolescent and young adult males found mixed results; two studies found that compared to their normal weight peers overweight and obese males were less likely to have ever had sexual intercourse [32, 33], and two studies found no relationship between BMI and sexual risk behaviors [29, 35]. Underweight youth, as well as overweight and obese youth, face the challenge and stress of being perceived as “different” in body size and image compared to their normal weight peers. Information about sexual risk behaviors and injection drug use among all youth who fall outside the normal range of BMI, including underweight, overweight, and obese adolescents, is needed to inform and better design interventions for HIV and other STD prevention efforts.

This study extends the current literature by examining associations between the full range of BMI (underweight, normal weight, overweight, and obese) and five sexual and nonsexual behaviors that may increase risk for acquiring HIV and other STDs, including sexual experience (i.e., ever having sexual intercourse), current sexual activity (i.e., having sexual intercourse during the past 3 months), having multiple sex partners, having sex without a condom, and injection drug use. Among the total student population, we examined associations of BMI with sexual experience and current sexual activity. Among currently sexually active students, we examined associations of BMI with having multiple sex partners, having sex without a condom, and injection drug use. The YRBS measures four behaviors which have been associated both with BMI and with sexual risk-taking and substance abuse, and which may act as mediators of those associations: suicidal thoughts [6, 7, 10–12], early sexual initiation [13–18], sexual abuse and nonconsensual sex [21–24, 26–28], and alcohol or drug use before sex [29, 30]. This study further extends the literature by examining, among currently sexually active students, whether these four behaviors (i.e., suicidal thoughts, early sexual initiation, sexual abuse and nonconsensual sex, and alcohol or drug use before sex) function as mediators by reducing the strength of significant associations between BMI and sexual risk behaviors (i.e., multiple sex partners and sex without a condom) and injection drug use.

## 2. Methods

### 2.1. Study Design

The national Youth Risk Behavior Survey (YRBS) has been conducted biennially, in odd-numbered years, since 1991. The surveys collected cross-sectional data from independently selected nationally representative samples of high school students on a wide range of priority health-risk behaviors. Each national YRBS used a similar three-stage probability sampling methodology which has been previously described [37]. A weighting factor was applied to each student record to adjust for the varying probabilities of selection at each stage of sampling, student nonresponse, and the oversampling of black and Hispanic students. The questionnaire contained approximately 98 items and was administered in the classroom during a regular class period by trained data collectors. Responses were recorded directly on computer-scannable questionnaire booklets or answer sheets. Student participation in the survey was anonymous and voluntary, and local procedures were used to obtain parental consent. The national YRBS has been reviewed and approved by an Institutional Review Board at the Centers for Disease Control and Prevention (CDC).

Data from the 2005, 2007, 2009, and 2011 national YRBS were combined to increase sample size sufficiently to allow for sex-stratified analysis of the associations between the full range of BMI and the sexual risk behaviors and injection drug use, among sexually active students. School response rates for these four surveys were 78%, 81%, 81%, and 81%, respectively [38–41]. Student response rates were 86%, 84%, 88%, and 87%, respectively. Overall response rates (defined as school response rate × student response rate) were 67%, 68%, 71%, and 71%, respectively. A small number of questionnaires (36 in 2005, 62 in 2007, 50 in 2009, and 78 in 2011) failed data edit checks leaving final sample sizes of 13,917, 14,041, 16,410, and 15,425, respectively. Thus, a total of 30,217 female students and 29,377 male students provided usable data during 2005–2011.

### 2.2. Measures

#### 2.2.1. Demographic Characteristics

Demographic characteristics included sex, race/ethnicity (non-Hispanic white, non-Hispanic black, Hispanic, and other), and grade (9th, 10th, 11th, and 12th).

#### 2.2.2. Sexual Risk Behaviors and Injection Drug Use

Sexual experience was assessed by asking, “Have you ever had sexual intercourse?” (coded yes versus no). Current sexual activity was assessed by asking, “During the past 3 months, with how many people have you had sexual intercourse?” (coded ≥1 versus 0). The following risk behaviors were assessed among currently sexually active students: “During your life, with how many people have you had sexual intercourse?” (coded ≥4 versus <4); “The last time you had sexual intercourse, did you or your partner use a condom?” (coded no versus yes); “During your life, how many times have you used a needle to inject any illegal drug into your body?” (coded ≥1 versus 0).

#### 2.2.3. Body Mass Index (BMI)

Self-reported height and weight (without shoes on) were used to calculate BMI, expressed as body weight in kilograms divided by the square of height in meters (kg/m^2^). Using age- and sex-specific reference data from growth charts produced by CDC, students with a BMI < 5th percentile were considered to be underweight; students with 5th percentile ≤ BMI < 85th percentile were considered normal weight; students with 85th percentile ≤ BMI < 95th percentile were considered overweight; and students with BMI ≥ 95th percentile were considered obese [42].

#### 2.2.4. Potential Mediators

Suicidal thoughts were assessed by asking, “During the past 12 months, did you ever seriously consider attempting suicide?” (coded yes versus no). Early sexual initiation was assessed by asking, “How old were you when you had sexual intercourse for the first time?” (coded <13 years versus ≥13 years). The YRBS assessed exposure to sexual abuse and nonconsensual sex by asking, “Have you ever been physically forced to have sexual intercourse when you did not want to?” (coded yes versus no). Finally, alcohol and drug use before sex was assessed by asking, “Did you drink alcohol or use drugs before you had sexual intercourse the last time?” (coded yes versus no).

### 2.3. Data Analysis

Data were weighted to provide national estimates and analyzed using SUDAAN version 10.0.1 (Research Triangle Institute, Research Triangle Park, NC). All analyses were stratified by sex. Differences, by sex, in BMI and HIV/STD-related risk behaviors were tested using Chi-square statistics. Sex-stratified multivariable logistic regression models were used to examine associations between BMI and HIV/STD-related risk behaviors, controlling for race/ethnicity and grade. Logistic regression models also were used to examine associations of potential mediators with BMI and with HIV/STD-related risk behaviors, controlling for demographic characteristics. Finally, we used logistic regression models to examine the associations of BMI with HIV/STD-related risk behaviors, controlling for demographics and potential mediators, to see if the inclusion of potential mediators reduced the strength of association between BMI and HIV/STD-related risk behaviors. Statistical tests were considered significant at *P* < 0.05, and odds ratios (OR) were considered significant if their 95% confidence intervals (CI) did not include 1.0.

## 3. Results

### 3.1. Prevalence of BMI Categories, Sexual Risk Behaviors, and Injection Drug Use

Approximately 69.4% (95% CI, 68.7–70.2) of students were normal weight, 2.4% (2.3–2.6) were underweight, 15.5% (15.0-16.0) were overweight, and 12.6% (12.1–13.2) were obese. Nearly half (47.0%, 45.5–48.4) of all students were sexually experienced, and a third (34.2%, 33.1–35.3) of students were currently sexually active. Among currently sexually active students, 37.9% (36.8–39.1) had 4 or more sex partners, 38.6% (37.5–39.7) did not use a condom at the last sexual intercourse, and 4.2% (3.8–4.6) had injected illegal drugs.

Compared to female students, male students were less likely to be normal weight (73.3% versus 65.8%, resp.) and more likely to be obese (9.3% versus 15.8%, resp.) (Table 1). Male students were more likely than female students to be sexually experienced but were less likely to be currently sexually active. Among currently sexually active students, male students were more likely than female students to have multiple sex partners and inject illegal drugs but were less likely to have sex without a condom.

### 3.2. Association of BMI with Sexual Risk Behaviors and Injection Drug Use

Among female students, underweight (OR = 0.71; 95% CI, 0.57–0.89) students were less likely than normal weight students to be sexually experienced. Among male students, both underweight (OR = 0.66; 0.53–0.83) and obese (OR = 0.87; 0.78–0.98) students were less likely than normal weight students to be sexually experienced (data not shown in tables).

Among female and male students, both underweight and obese students were less likely than normal weight students to be currently sexually active (female students, OR = 0.75 and OR = 0.80, resp.; male students, OR = 0.61 and OR = 0.78, resp.) (Table 2).

Among currently sexually active female students, compared to normal weight females, overweight (OR = 1.45) and obese (OR = 1.59) females were more likely to have multiple sex partners; underweight (OR = 1.91), overweight (OR = 1.18), and obese (OR = 1.30) females were more likely to have sex without a condom; and obese (OR = 1.98) females were more likely to inject illegal drugs (Table 2).

Among currently sexually active male students, compared to normal weight males, overweight (OR = 1.19) males were more likely to have sex without a condom, and obese (OR = 1.42) males were more likely to inject illegal drugs (Table 2). The association between obesity and multiple sex partners approached statistical significance (OR = 1.17, *P* = 0.052).

### 3.3. Potential Mediators

Among currently sexually active female students, compared to normal weight females, overweight (OR = 1.24) and obese (OR = 1.52) females were more likely to have suicidal thoughts; overweight (OR = 1.47) and obese (OR = 2.12) females were more likely to have had early sexual initiation; and obese (OR = 1.37) females were more likely to have experienced sexual abuse and nonconsensual sex (Table 3). Alcohol or drug use before sex was not associated with BMI. All four potential mediators were associated with multiple sex partners, no condom use, and injection drug use.

Among currently sexually active male students, compared to normal weight males, obese (OR = 1.45) males were more likely to have suicidal thoughts; overweight (OR = 1.32) and obese (OR = 1.54) males were more likely to have had early sexual initiation; and underweight (OR = 0.59) males were less likely to have used alcohol or drugs before sex (Table 3). Exposure to sexual abuse and nonconsensual sex was not associated with BMI. All four potential mediators were associated with multiple sex partners, no condom use, and injection drug use.

A final set of sex-stratified logistic regression models was run to assess the effect of inclusion of potential mediators on the associations of BMI with HIV/STD-related risk behaviors (i.e., for comparison to ORs in Table 2). In regression models for sexually active female students (Table 4), use of alcohol or drugs before sex was not included because it was not associated with BMI. In regression models for sexually active male students (Table 5), exposure to sexual abuse and nonconsensual sex was not included because it was not associated with BMI.

Among currently sexually active female students, compared to normal weight females, overweight (OR = 1.39) and obese (OR = 1.39) females were more likely to have multiple sex partners and underweight (OR = 1.99) and obese (OR = 1.23) females were more likely to have sex without a condom (Table 4). Compared to ORs in Table 2, the strength of association between overweight and obesity and sexual risk behaviors and injection drug use consistently decreased, and in some instances (no condom use among overweight females and injection drug use among obese females) ceased to be statistically significant.

Among currently sexually active male students, compared to normal weight males, overweight (OR = 1.19) males were more likely to have sex without a condom (Table 5). Compared to ORs in Table 2, the strength of association between overweight and not using a condom did not change; however, the strength of association between obesity and injection drug use decreased and ceased to be statistically significant. The strength of association between obesity and multiple sex partners, which was marginally significant (OR = 1.17, *P* = 0.052) in Table 2, decreased and was no longer significant (OR = 1.02, *P* = 0.81).

## 4. Discussion

BMI was related to both protective and risky sexual behaviors and injection drug use among high school students. BMI was protective for current sexual activity among underweight and obese females and males as compared to their normal weight peers. However, among currently sexually active students, overweight and obese female students were more likely than normal weight female students to have multiple sex partners and to have sex without a condom; underweight female students also were more likely to have sex without a condom. Among sexually active male students, overweight was significantly associated with increased likelihood of having sex without a condom and obesity was marginally (*P* = 0.052) associated with having multiple sex partners. Among sexually active students, 2.7% of female students and 5.6% of male students reported injecting an illegal drug into their body. Obese sexually active students were more likely than normal weight sexually active students to inject illegal drugs.

Our study is one of the few studies to have examined sexual risk behaviors among currently sexually active students. Our findings are consistent with studies that have found an association between BMI and sexual initiation among both female and male students [29, 31–34] and also support prior findings that sexually experienced overweight and obese adolescents may engage in more risky sexual behavior more than their normal weight peers [29, 31, 35, 36]. Studies including related constructs such as body image, perceived weight status, and measures of attractiveness also support findings of increased sexual risk behaviors among adolescents who perceive themselves to be less attractive, overweight, or obese or whose perception of weight is not congruent with their BMI relative to their normal weight peers [32–34, 36, 43, 44].

Our examination of potential mediating factors suggests that, among currently sexually active female and male students, suicidal thoughts and early sexual initiation mediate in part the relationships between BMI and multiple sex partners, lack of condom use, and injection drug use. Also acting as potential mediators were sexual abuse and nonconsensual sex (among female students) and alcohol or drug use before sexual intercourse (among male students), but those mediators acted less strongly than suicidal thoughts and early sexual initiation. Among sexually active female students, the inclusion of mediators in regression models resulted in a weakening of the strength of association between overweight and obesity and all three HIV/STD-related risk behaviors, and in some instances (e.g., no condom use among overweight females and injection drug use among obese females) these associations ceased to be statistically significant; however, the association between underweight and lack of condom use remained significant. Among sexually active male students, the inclusion of mediators in the regression models did not change the strength of association between overweight and lack of condom use; however, the strength of association between obesity and injection drug use decreased and ceased to be statistically significant, and the strength of association between obesity and multiple sex partners, which was marginally significant, decreased and was no longer significant. These findings are consistent with previous studies that suggest that suicidal thoughts and behaviors [6, 7, 10–12] and early sexual initiation [13–18] may be associated with obesity, risky sexual behavior, and substance abuse. Suicidal thoughts and early sexual initiation may be strong mediating factors of the association between obesity and injection drug use, as this association becomes statistically insignificant after controlling for these factors. This finding is consistent with a recent study showing an exceptionally strong association between injection drug use and suicide attempts among adolescents [12].

Prior research on BMI and sexual risk behavior suggests that these relationships are part of a constellation of risk behaviors that can signal disproportionate risk for adverse health outcomes among some underweight, overweight, and obese adolescents. Although BMI may be a protective factor for decreased likelihood of sexual experience and current sexual activity, such “protection” may reflect social stigma related to perceived weight and attractiveness. Overweight and obesity are associated with a greater likelihood of peer rejection and marginalization [45, 46], increased likelihood of victimization [47], less likelihood of dating and of engaging in intimate relationships [32–34, 39, 47], and increased social stigma [33] relative to normal weight peers. These factors may also contribute to the occurrence of sexual risk behaviors among sexually active youth.

Recent studies have demonstrated an increasing trend in the prevalence of youth at the highest levels of obesity (i.e., those whose BMI is greater than the standard obesity cutoff of BMI ≥ 95th percentile) [48, 49]. To examine whether the pattern of associations was different among the very heaviest youth, we repeated our analysis using an obesity cutoff of BMI ≥ 97th percentile. We found that the patterns of association of sexual risk-taking and injection drug use with obesity at the BMI ≥ 97th percentile level were identical to the patterns of association at the standard obesity cutoff of BMI ≥ 95th percentile (Table 2). For all significant associations, the odds ratios were stronger at the BMI ≥ 97th percentile level than the BMI ≥ 95th percentile level (data not shown).

This study is subject to several limitations. First, these data apply only to youths who attend school and therefore are not representative of all people in this age group. Nationwide, in 2009, of people aged 16-17, approximately 4% were not enrolled in a high school program and had not completed high school [50]. Second, the extent of underreporting or overreporting of self-reported behaviors cannot be determined, although the survey questions demonstrate good test-retest reliability [51]. Third, the data are cross-sectional; therefore, causality and directionality of associations cannot be determined. Fourth, it is possible that the prevalence of risk behaviors may have changed significantly during 2005–2011 such that differences in these associations over time are being masked. However, we found no significant changes over this time period in the prevalence of obesity, multiple sex partners, not using a condom, or injection drug use among currently sexually active male or female students. Fifth, our measure of BMI was based on self-reported rather than measured height and weight. However, self-reported and measured BMI are highly correlated, and our goal was simply to partition students into body weight categories based on their relative BMI. Finally, we provided no data on rates of HIV infection and STDs, and while it is possible that BMI is associated with these clinical outcomes we were not able to examine that hypothesis in this study. It is possible that BMI is associated with factors we did not measure that work to lower rates of HIV infection and STDs among youth. Also, for example, the lower rates of sexual experience and current sexual activity we found among obese students may actually result in lower rates of HIV infection and STDs among the total population of obese students even though, among sexually active students, obese students are more likely than normal weight students to engage in some sexual risk behaviors and injection drug use. What we have been able to show is that among currently sexually active students, compared to normal weight students, having a BMI that falls outside the normal range is associated with multiple sex partners, sex without a condom, and injection drug use, all of which may act to increase risk of acquiring HIV and other STDs. This has important implications for school health professionals and programs designed to prevent HIV infection and STDs among high school students.

## 5. Conclusions

Among sexually active high school students, having a BMI outside the normal range was associated with sexual risk behaviors and injection drug use which may act to increase risk for HIV infection and STDs. Community-based pediatric, adolescent and bariatric health professionals, and school-based health and mental health professionals involved in HIV and STD prevention programs may want to consider that sexually active students who are underweight, overweight, or obese may have a greater likelihood of engaging in some risky sexual behaviors and injection drug use than their normal weight peers and may merit targeted messaging around healthy sexual behaviors and substance use. In addition, programs to ensure a healthy school environment may want to strive to decrease stigma based on students' perceived weight and body size. Increasing opportunities for underweight, overweight, and obese students to connect with adults and peers at school may help to mitigate the greater likelihood of peer rejection and marginalization, social stigmatization, victimization, and suicidal thoughts and behaviors experienced by students with body weight issues relative to their normal weight peers. In addition, it may help if classroom activities are inclusive of all students, regardless of weight and size, and school policies to prevent bullying and harassment include students with weight issues [52].

## Figures and Tables

**Table 1:** Prevalence of BMI categories and HIV/STD-related risk behaviors, by sex among high school students, United States, 2005–2011.

Subgroups	Female students, *N* = 30217			Male sudents, *N* = 29377			Chi-square
	*n*	%	(95% CI)	*N*	%	(95% CI)	*P* value
BMI category							
Normal	20336	73.3	(72.3–74.2)	17825	65.8	(64.8–66.8)	*P* = 0.0000
Underweight	603	2.2	(2.0–2.5)	750	2.7	(2.4–3.0)	
Overweight	4614	15.3	(14.7–15.9)	4421	15.7	(15.1–16.3)	
Obese	2892	9.3	(8.7–9.8)	4678	15.8	(15.0–16.7)	
HIV-related behaviors							
Sexual experience^a^	28051	45.7	(44.3–47.2)	26661	48.2	(46.3–50.0)	*P* = 0.0023
Current sexual activity^b^	28014	35.0	(33.8–36.2)	26539	33.3	(32.0–34.7)	*P* = 0.0176
Multiple sex partners^c^	10406	30.7	(29.4–32.0)	9934	45.3	(43.7–47.0)	*P* = 0.0000
No condom use^d^	10284	45.5	(44.1–46.9)	9744	31.5	(30.0–33.1)	*P* = 0.0000
Injection drug use^e^	10245	2.7	(2.4–3.2)	9791	5.6	(5.0–6.3)	*P* = 0.0000

BMI: body mass index = weight [kg]/height [m]^2^ (based on self-reported height and weight, using age- and sex-specific percentiles from growth charts developed by Centers for Disease Control and Prevention); normal = 5th percentile ≤ BMI < 85th percentile; underweight = BMI < 5th percentile; overweight = 85th percentile ≤ BMI < 95th percentile; and obese = BMI ≥ 95th percentile. HIV: human immunodeficiency virus. STD: sexually transmitted disease. CI: confidence interval.

^a^Ever had sexual intercourse.

^b^Had sexual intercourse during the 3 months before the survey.

^c^Had sexual intercourse with four or more persons during their lifetime (among students who were currently sexually active).

^d^Did not use a condom during last sexual intercourse (among students who were currently sexually active).

^e^Ever used a needle to inject any illegal drug into their body (among students who were currently sexually active).

**Table 2:** Prevalence and adjusted odds ratios for HIV/STD-related risk behaviors by BMI, among female and male high school students, United States, 2005–2011.

Subgroups	Current sexual activity^a^			Multiple sex partners^b^			No condom use^c^			Injection drug use^d^		
	%	OR	(95% CI)	%	OR	(95% CI)	%	OR	(95% CI)	%	OR	(95% CI)
*Female *												
BMI category												
Normal	35.8	1.00	(Referent)	28.4	1.00	(Referent)	44.0	1.00	(Referent)	2.3	1.00	(Referent)
Underweight	31.1	**0.75**	**(0.60–0.94)**	37.6	1.44	(0.942–2.19)	61.4	**1.91**	**(1.26–2.89)**	3.1	1.62	(0.64–4.15)
Overweight	34.8	0.94	(0.85–1.04)	36.3	**1.45**	**(1.22–1.71)**	47.7	**1.18**	**(1.01–1.36)**	2.0	0.86	(0.51–1.44)
Obese	31.9	**0.80**	**(0.71–0.89)**	39.1	**1.59**	**(1.32–1.91)**	49.3	**1.30**	**(1.07–1.57)**	4.2	**1.98**	**(1.15–3.41)**

*Male *												
BMI category												
Normal	34.0	1.00	(Referent)	43.2	1.00	(Referent)	30.0	1.00	(Referent)	4.4	1.00	(Referent)
Underweight	23.6	**0.61**	**(0.49–0.76)**	38.2	0.79	(0.51–1.23)	35.8	1.28	(0.87–1.91)	5.3	0.95	(0.44–2.02)
Overweight	34.4	1.04	(0.95–1.14)	47.3	1.15	(0.97–1.38)	33.8	**1.19**	**(1.04–1.37)**	4.6	1.03	(0.73–1.45)
Obese	29.4	**0.78**	**(0.69–0.87)**	47.7	1.17	(1.00–1.37)	32.6	1.11	(0.94–1.32)	6.2	**1.42**	**(1.03–1.96)**

BMI: body mass index = weight [kg]/height [m]^2^ (based on self-reported height and weight, using age- and sex-specific percentiles from growth charts developed by Centers for Disease Control and Prevention); normal = 5th percentile ≤ BMI < 85th percentile; underweight = BMI < 5th percentile; overweight = 85th percentile ≤ BMI < 95th percentile; and obese = BMI ≥ 95th percentile. HIV: human immunodeficiency virus. STD: sexually transmitted disease. OR: odds ratio, adjusted for race/ethnicity and grade. CI: confidence interval.

^a^Had sexual intercourse during the 3 months before the survey.

^b^Had sexual intercourse with four or more persons during their lifetime (among students who were currently sexually active).

^c^Did not use a condom during last sexual intercourse (among students who were currently sexually active).

^d^Ever used a needle to inject any illegal drug into their body (among students who were currently sexually active).

**Table 3:** Associations of BMI and HIV/STD-related risk behaviors with potential mediators, among currently sexually active^a^ high school students, United States, 2005–2011.

Subgroups	Suicidal thoughts^b^		Early sexual initiation^c^		Sexual abuse and nonconsensual sex^d^		Alcohol or drug use before sex^e^	
	OR	(95% CI)	OR	(95% CI)	OR	(95% CI)	OR	(95% CI)
*Female *								
BMI category								
Normal (referent)	1.0	—	1.0	—	1.0	—	1.0	—
Underweight	0.92	(0.60–1.43)	1.34	(0.63–2.82)	1.45	(0.89–2.36)	1.21	(0.75–1.95)
Overweight	**1.24**	**(1.05–1.48)**	**1.47**	**(1.15–1.89)**	1.17	(0.96–1.41)	1.06	(0.86–1.31)
Obese	**1.52**	**(1.23–1.87)**	**2.12**	**(1.60-2.81)**	**1.37**	**(1.12–1.67)**	1.23	(0.96–1.57)
Multiple sex partners^f^								
No (referent)	1.0	—	1.0	—	1.0	—	1.0	—
Yes	**1.80**	**(1.60–2.03)**	**6.33**	**(5.06–7.93)**	**2.80**	**(2.42–3.23)**	**2.47**	**(2.17–2.80)**
No condom use^g^								
No (referent)	1.0	—	1.0	—	1.0	—	1.0	—
Yes	**1.43**	**(1.29–1.60)**	**1.96**	**(1.54–2.50)**	**1.62**	**(1.43–1.83)**	**1.25**	**(1.10–1.42)**
Injection drug use^h^								
No (referent)	1.0	—	1.0	—	1.0	—	1.0	—
Yes	**3.87**	**(2.86–5.24)**	**6.15**	**(4.01–9.43)**	**4.57**	**(3.21–6.51)**	**5.15**	**(3.70–7.17)**

*Male *								
BMI category								
Normal (referent)	1.0	—	1.0	—	1.0	—	1.0	—
Underweight	1.44	(0.85–2.44)	0.92	(0.54–1.57)	0.86	(0.45–1.67)	**0.59**	**(0.39–0.88)**
Overweight	0.89	(0.72–1.09)	**1.32**	**(1.10–1.58)**	0.94	(0.72–1.23)	1.09	(0.91–1.30)
Obese	**1.45**	**(1.13-1.85)**	**1.54**	**(1.26–1.89)**	1.24	(0.92–1.67)	1.13	(0.95–1.35)
Multiple sex partners^f^								
No (referent)	1.0	—	1.0	—	1.0	—	1.0	—
Yes	**1.43**	**(1.23–1.66)**	**7.78**	**(6.47–9.36)**	**4.00**	**(3.19–5.00)**	**3.20**	**(2.84–3.59)**
No condom use^g^								
No (referent)	1.0	—	1.0	—	1.0	—	1.0	—
Yes	**2.00**	**(1.71–2.34)**	**1.58**	**(1.37–1.83)**	**2.58**	**(2.15–3.10)**	**1.34**	**(1.16–1.54)**
Injection drug use^h^								
No (referent)	1.0	—	1.0	—	1.0	—	1.0	—
Yes	**6.55**	**(5.20–8.25)**	**7.82**	**(5.96–10.2)**	**11.2**	**(8.63–14.7)**	**8.17**	**(6.40–10.4)**

BMI: body mass index = weight [kg]/height [m]^2^ (based on self-reported height and weight, using age- and sex-specific percentiles from growth charts developed by Centers for Disease Control and Prevention); normal = 5th percentile ≤ BMI < 85th percentile; underweight = BMI < 5th percentile; overweight = 85th percentile ≤ BMI < 95th percentile; and obese = BMI ≥ 95th percentile. HIV: human immunodeficiency virus. STD: sexually transmitted disease. CI: confidence interval. OR: odds ratio, adjusted for race/ethnicity and grade.

^a^Had sexual intercourse during the 3 months before the survey.

^b^Seriously considered attempting suicide during the past 12 months.

^c^Had first sexual intercourse before age of 13.

^d^Ever been physically forced to have sexual intercourse when they did not want to.

^e^Used alcohol or drugs before last sexual intercourse.

^f^Had sexual intercourse with four or more persons during their lifetime (among students who were currently sexually active).

^g^Did not use a condom during last sexual intercourse (among students who were currently sexually active).

^h^Ever used a needle to inject any illegal drug into their body (among students who were currently sexually active).

**Table 4:** Prevalence and adjusted odds ratios for HIV/STD-related risk behaviors among currently sexually active^a^ female high school students, United States, 2005–2011.

Regression model	Multiple sex partners^b^			No condom use^c^			Injection drug use^d^		
	%	OR	(95% CI)	%	OR	(95% CI)	%	OR	(95% CI)
BMI category									
Normal	28.4	1.00	(Referent)	44.0	1.00	(Referent)	2.3	1.00	(Referent)
Underweight	37.6	1.28	(0.84–1.97)	61.4	**1.99**	**(1.36–2.91)**	3.1	1.65	(0.67–4.10)
Overweight	36.3	**1.39**	**(1.17–1.65)**	47.7	1.15	(0.98–1.33)	2.0	0.73	(0.42–1.26)
Obese	39.1	**1.39**	**(1.13–1.71)**	49.3	**1.23**	**(1.01–1.49)**	4.2	1.55	(0.88–2.74)
Race/ethnicity									
White	29.8	1.00	(Referent)	45.2	1.00	(Referent)	2.5	1.00	(Referent)
Black	35.8	**1.31**	**(1.12–1.54)**	43.2	0.92	(0.79–1.09)	1.1	**0.35**	**(0.17–0.70)**
Hispanic	27.5	**0.85**	**(0.74–0.99)**	49.3	**1.19**	**(1.04–1.36)**	4.5	1.27	(0.81–2.01)
Other	33.2	1.12	(0.87–1.43)	44.0	0.94	(0.74–1.19)	4.2	0.96	(0.53–1.73)
Grade									
9th	25.0	1.00	(Referent)	38.6	1.00	(Referent)	5.7	1.00	(Referent)
10th	26.3	1.23	(0.98–1.54)	40.7	1.18	(0.99–1.41)	3.1	**0.57**	**(0.35–0.92)**
11th	30.7	**1.73**	**(1.40–2.14)**	44.5	**1.43**	**(1.21–1.68)**	2.2	**0.52**	**(0.33–0.84)**
12th	35.9	**2.44**	**(1.95–3.07)**	52.2	**2.02**	**(1.71–2.40)**	1.3	**0.37**	**(0.22–0.61)**
Suicidal thoughts^e^									
No	28.0	1.00	(Referent)	43.6	1.00	(Referent)	1.5	1.00	(Referent)
Yes	39.0	**1.44**	**(1.25–1.66)**	50.8	**1.27**	**(1.13–1.43)**	6.5	**2.61**	**(1.81–3.78)**
Early sexual initiation^f^									
No	28.2	1.00	(Referent)	44.7	1.00	(Referent)	2.0	1.00	(Referent)
Yes	67.6	**4.79**	**(3.68–6.23)**	57.9	**1.72**	**(1.35–2.18)**	13.5	**3.59**	**(2.26–5.69)**
Sexual abuse and nonconsensual sex^g^									
No	26.0	1.00	(Referent)	43.1	1.00	(Referent)	1.6	1.00	(Referent)
Yes	48.0	**2.23**	**(1.89–2.64)**	54.3	**1.43**	**(1.25–1.63)**	7.2	**2.67**	**(1.74–4.09)**

BMI: body mass index = weight [kg]/height [m]^2^ (based on self-reported height and weight, using age- and sex-specific percentiles from growth charts developed by Centers for Disease Control and Prevention); normal = 5th percentile ≤ BMI < 85th percentile; underweight = BMI < 5th percentile; overweight = 85th percentile ≤ BMI < 95th percentile; obese = BMI ≥ 95th percentile. HIV: human immunodeficiency virus. STD: sexually transmitted disease. OR: odds ratio, adjusted for other variables in the model. CI: confidence interval.

^a^Had sexual intercourse during the 3 months before the survey.

^b^Had sexual intercourse with four or more persons during their lifetime (among students who were currently sexually active).

^c^Used a condom during last sexual intercourse (among students who were currently sexually active).

^d^Ever used a needle to inject any illegal drug into their body (among students who were currently sexually active).

^e^Seriously considered attempting suicide during the past 12 months.

^f^Had first sexual intercourse before age of 13.

^g^Ever been physically forced to have sexual intercourse when they did not want to.

**Table 5:** Prevalence and adjusted odds ratios for HIV/STD-related risk behaviors among currently sexually active^a^ male high school students, United States, 2005–2011.

Regression model	Multiple sex partners^b^			No condom use^c^			Injection drug use^d^		
	%	OR	(95% CI)	%	OR	(95% CI)	%	OR	(95% CI)
BMI category									
Normal	43.2	1.00	(Referent)	30.0	1.00	(Referent)	4.4	1.00	(Referent)
Underweight	38.2	0.83	(0.52–1.33)	35.8	1.25	(0.84–1.86)	5.3	0.91	(0.45–1.82)
Overweight	47.3	1.08	(0.90–1.30)	33.8	**1.19**	**(1.04–1.38)**	4.6	0.85	(0.57–1.26)
Obese	47.7	1.02	(0.87–1.20)	32.6	1.03	(0.87–1.23)	6.2	1.02	(0.72–1.45)
Race/ethnicity									
White	36.2	1.00	(Referent)	31.5	1.00	(Referent)	5.2	1.00	(Referent)
Black	66.0	**3.28**	**(2.72–3.96)**	25.8	**0.76**	**(0.64–0.90)**	3.5	**0.46**	**(0.29–0.73)**
Hispanic	48.9	**1.74**	**(1.50–2.01)**	35.2	**1.18**	**(1.01–1.37)**	6.9	1.16	(0.80–1.70)
Other	50.7	**1.59**	**(1.19–2.12)**	37.4	1.20	(0.90–1.61)	10.0	1.11	(0.70–1.77)
Grade									
9th	45.6	1.00	(Referent)	27.7	1.00	(Referent)	6.9	1.00	(Referent)
10th	46.3	**1.43**	**(1.15–1.79)**	27.8	1.16	(0.94–1.43)	6.1	1.52	(0.99–2.34)
11th	42.9	**1.49**	**(1.21–1.84)**	32.1	**1.43**	**(1.16–1.77)**	5.6	1.51	(0.99–2.29)
12th	46.4	**1.94**	**(1.59–2.36)**	36.1	**1.73**	**(1.38–2.16)**	4.1	1.21	(0.80–1.85)
Suicidal thoughts^e^									
No	44.4	1.00	(Referent)	28.9	1.00	(Referent)	3.1	1.00	(Referent)
Yes	50.8	1.05	(0.88–1.26)	45.6	**1.85**	**(1.57–2.18)**	18.5	**4.17**	**(3.08–5.62)**
Early sexual initiation^f^									
No	36.4	1.00	(Referent)	30.2	1.00	(Referent)	2.9	1.00	(Referent)
Yes	81.4	**6.83**	**(5.61–8.31)**	36.7	**1.38**	**(1.17–1.61)**	16.3	**5.14**	**(3.69–7.17)**
Alcohol or drug use before sex^g^									
No	38.6	1.00	(Referent)	29.6	1.00	(Referent)	2.1	1.00	(Referent)
Yes	63.5	**2.72**	**(2.38–3.11)**	36.8	1.15	(0.99–1.33)	15.2	**5.17**	**(3.94–6.79)**

BMI: body mass index = weight [kg]/height [m]^2^ (based on self-reported height and weight, using age- and sex-specific percentiles from growth charts developed by Centers for Disease Control and Prevention); normal = 5th percentile ≤ BMI < 85th percentile; underweight = BMI < 5th percentile; overweight = 85th percentile ≤ BMI < 95th percentile; and obese = BMI ≥ 95th percentile. HIV: human immunodeficiency virus. STD: sexually transmitted disease. OR: odds ratio, adjusted for other variables in the model. CI: confidence interval.

^a^Had sexual intercourse during the 3 months before the survey.

^b^Had sexual intercourse with four or more persons during their lifetime (among students who were currently sexually active).

^c^Used a condom during last sexual intercourse (among students who were currently sexually active).

^d^Ever used a needle to inject any illegal drug into their body (among students who were currently sexually active).

^e^Seriously considered attempting suicide during the past 12 months.

^f^Had first sexual intercourse before age of 13.

^g^Used alcohol or drugs before last sexual intercourse.

## References

[1] Ogden CL, Carroll MD, Kit BK, Flegal KM (2012). Prevalence of obesity and trends in body mass index among US children and adolescents, 1999–2010. *The Journal of the American Medical Association*.

[2] Dietz WH (1998). Health consequences of obesity in youth: childhood predictors of adult disease. *Pediatrics*.

[3] Freedman DS, Dietz WH, Srinivasan SR, Berenson GS (1999). The relation of overweight to cardiovascular risk factors among children and adolescents: the Bogalusa heart study. *Pediatrics*.

[4] Strauss RS (2000). Childhood obesity and self-esteem. *Pediatrics*.

[5] Kalarchian MA, Marcus MD (2012). Psychiatric comorbidity of childhood obesity. *International Review of Psychiatry*.

[6] Falkner NH, Neumark-Sztainer D, Story M, Jeffery RW, Beuhring T, Resnick MD (2001). Social, educational, and psychological correlates of weight status in adolescents. *Obesity Research*.

[7] Eaton DK, Lowry R, Brener ND, Galuska DA, Crosby AE (2005). Associations of body mass index and perceived weight with suicide ideation and suicide attempts among US high school students. *Archives of Pediatrics and Adolescent Medicine*.

[8] Centers for Disease Control and Prevention (2012). Vital signs: HIV infection, testing, and risk behaviors among youths—United States. *Morbidity and Mortality Weekly Report*.

[9] Satterwhite CL, Torrone E, Meites E (2013). Sexually transmitted infections among US women and men: prevalence and incidence estimates, 2008. *Sexually Transmitted Diseases*.

[10] Lehrer JA, Shrier LA, Gortmaker S, Buka S (2006). Depressive symptoms as a longitudinal predictor of sexual risk behaviors among US middle and high school students. *Pediatrics*.

[11] Ores LDC, Quevedo LDA, Jansen K (2012). Suicide risk and health risk behavior among youth between the ages of 18 and 24 years: a descriptive study. *Cadernos de Saude Publica*.

[12] Lowry R, Crosby A, Brener N, Kann L (2014). Suicidal thoughts and attempts among U.S. High school students: trends and associated health-risk behaviors, 1991–2011. *Journal of Adolescent Health*.

[13] Wang Y (2002). Is obesity associated with early sexual maturation? A comparison of the association in American boys versus girls. *Pediatrics*.

[14] Currie C, Ahluwalia N, Godeau E, Nic Gabhainn S, Due P, Currie DB (2012). Is obesity at individual and national level associated with lower age at menarche? Evidence from 34 countries in the health behaviour in school-aged children study. *Journal of Adolescent Health*.

[15] Johnson W, Stovitz SD, Choh AC, Czerwinski SA, Towne B, Demerath EW (2012). Patterns of linear growth and skeletal maturation from birth to 18 years of age in overweight young adults. *International Journal of Obesity*.

[16] Madkour AS, Farhat T, Halpern CT, Godeau E, Gabhainn SN (2010). Early adolescent sexual initiation as a problem behavior: a comparative study of five nations. *Journal of Adolescent Health*.

[17] Sandfort TGM, Orr M, Hirsch JS, Santelli J (2008). Long-term health correlates of timing of sexual debut: results from a national US study. *The American Journal of Public Health*.

[18] Harrison A, Cleland J, Gouws E, Frohlich J (2005). Early sexual debut among young men in rural South Africa: heightened vulnerability to sexual risk?. *Sexually Transmitted Infections*.

[19] Kim DS, Kim HS (2010). Early initiation of alcohol drinking, cigarette smoking, and sexual intercourse linked to suicidal ideation and attempts: findings from the 2006 Korean youth risk behavior survey. *Yonsei Medical Journal*.

[20] Maniglio R (2009). The impact of child sexual abuse on health: a systematic review of reviews. *Clinical Psychology Review*.

[21] Wilson DR (2010). Health consequences of childhood sexual abuse. *Perspectives in Psychiatric Care*.

[22] Raj A, Silverman JG, Amaro H (2000). The relationship between sexual abuse and sexual risk among high school students: findings from the 1997 Massachusetts youth risk behavior survey. *Maternal and Child Health Journal*.

[23] Homma Y, Wang N, Saewyc E, Kishor N (2012). The relationship between sexual abuse and risky sexual behavior among adolescent boys: a meta-analysis. *Journal of Adolescent Health*.

[24] Molnar BE, Buka SL, Kessler RC (2001). Child sexual abuse and subsequent psychopathology: results from the national comorbidity survey. *The American Journal of Public Health*.

[25] Bruffaerts R, Demyttenaere K, Borges G (2010). Childhood adversities as risk factors for onset and persistence of suicidal behaviour. *The British Journal of Psychiatry*.

[26] Shin SH, Miller DP (2012). A longitudinal examination of childhood maltreatment and adolescent obesity: results from the national longitudinal study of adolescent health (AddHealth) study. *Child Abuse and Neglect*.

[27] Midei AJ, Matthews KA (2011). Interpersonal violence in childhood as a risk factor for obesity: a systematic review of the literature and proposed pathways. *Obesity Reviews*.

[28] Gustafson TB, Sarwer DB (2004). Childhood sexual abuse and obesity. *Obesity Reviews*.

[29] Ratcliff MB, Jenkins TM, Reiter-Purtill J, Noll JG, Zeller MH (2011). Risk-taking behaviors of adolescents with extreme obesity: normative or not?. *Pediatrics*.

[30] Tucker JS, Ryan GW, Golinelli D (2012). Substance use and other risk factors for unprotected sex: results from an event-based study of homeless youth. *AIDS and Behavior*.

[31] Averett S, Corman H, Reichman N (2010). *Effects of Overweight on Risky Sexual Behavior of Adolescent Girls*.

[32] Cawley J, Joyner K, Sobal J (2006). Size matters: the influence of adolescents' weight and height on dating and sex. *Rationality and Society*.

[33] Cheng YH-A, Landale NS (2013). Teen overweight, weight stigma and intimate relationship development from adolescent to young adulthood. *Working Paper*.

[34] Heiland F, Ali M (2010). Racial differences in the influence of female adolescent’s body size on dating and sex. *CUNY Institute for Demography Working Paper*.

[35] Eisenberg ME, Neumark-Sztainer D, Lust KD (2005). Weight-related issues and high-risk sexual behaviors among college students. *Journal of American College Health*.

[36] Villers M Sexual behavior in obese and overweight adolescent female students.

[37] Brener ND, Kann L, Shanklin S (2013). Methodology of the youth risk behavior surveillance system—2013. *Morbidity and Mortality Weekly Report: Recommendations and Reports*.

[38] Centers for Disease Control and Prevention (2012). Youth risk behavior surveillance—United States, 2011. *Morbidity and Mortality Weekly Report: Surveillance Summaries*.

[39] Centers for Disease Control and Prevention (2009). Youth risk behavior surveillance—United States. *Morbidity and Mortality Weekly Report: Surveillance Summaries*.

[40] Centers for Disease Control and Prevention (2008). Youth risk behavior surveillance—United States, 2007. *Morbidity and Mortality Weekly Report: Surveillance Summaries*.

[41] Centers for Disease Control and Prevention (2006). Youth risk behavior surveillance—United States, 2005. *Morbidity and Mortality Weekly Report: Surveillance Summaries*.

[42] Barlow SE (2007). Expert committee recommendations regarding the prevention, assessment, and treatment of child and adolescent overweight and obesity: summary report. *Pediatrics*.

[43] Halpern CT, Udry JR, Campbell B, Suchindran C (1999). Effects of body fat on weight concerns, dating, and sexual activity: a longitudinal analysis of black and white adolescent girls. *Developmental Psychology*.

[44] Akers AY, Lynch CP, Gold MA (2009). Exploring the relationship among weight, race, and sexual behaviors among girls. *Pediatrics*.

[45] Zeller MH, Reiter-Purtill J, Ramey C (2008). Negative peer perceptions of obese children in the classroom environment. *Obesity*.

[46] Strauss RS, Pollack HA (2003). Social marginalization of overweight children. *Archives of Pediatrics and Adolescent Medicine*.

[47] Pearce MJ, Boergers J, Prinstein MJ (2002). Adolescent obesity, overt and relational peer victimization, and romantic relationships. *Obesity Research*.

[48] Ogden CL, Carroll MD, Curtin LR, Lamb MM, Flegal KM (2010). Prevalence of high body mass index in US children and adolescents, 2007-2008. *The Journal of the American Medical Association*.

[49] Skinner AC, Skelton JA (2014). Prevalence and trends in obesity among children in the United States, 1999–2012. *JAMA Pediatrics*.

[50] US Department of Education Common core of data. http://nces.ed.gov/ccd.

[51] Brener ND, Kann L, McManus T, Kinchen SA, Sundberg EC, Ross JG (2002). Reliability of the 1999 youth risk behavior survey questionnaire. *Journal of Adolescent Health*.

[52] Centers for Disease Control and Prevention (2011). School health guidelines to promote healthy eating and physical activity. *Morbidity and Mortality Weekly Report: Recommendations and Reports*.

